# A 2:1 co-crystal of 3,5-di­bromo-4-cyano­benzoic acid and anthracene

**DOI:** 10.1107/S2056989017014815

**Published:** 2017-10-20

**Authors:** Wayland E. Noland, Janel L. Rieger, Zoe H. Tu, Kenneth J. Tritch

**Affiliations:** a Department of Chemistry, University of Minnesota, 207 Pleasant St SE, Minneapolis, MN 55455, USA

**Keywords:** crystal structure, co-crystal, carb­oxy­lic acid, N⋯Br contacts

## Abstract

The title co-crystal, C_8_H_3_Br_2_NO_2_·0.5C_14_H_10_, was self-assembled from a 2:1 mixture of the components in slowly evaporating di­chloro­methane. The mol­ecules adopt a sheet structure parallel to (1-12) in which carb­oxy hydrogen-bonded dimers and anthracene mol­ecules stagger in both dimensions. Within the sheets, six individual cyano acid mol­ecules surround each anthracene mol­ecule. Cyano acid mol­ecules form one of the two possible *R*_2_^2^(10) rings between neighboring cyano and bromo groups. Compared to the di­chloro analog [Britton (2012 ▸). *J. Chem. Crystallogr.* **42**, 851–855], the dihedral angle between the best-fit planes of acid and anthracene mol­ecules has decreased from 7.1 to 0.9 (2)°.

## Abstract 2

The title cyano acid forms a honeycomb-like sheet structure, six mol­ecules circumscribing anthracene, with carb­oxy hydrogen-bonded dimers linked by half of the possible 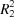
(10) CN⋯Br rings.

## Chemical context   

**Figure 1:**
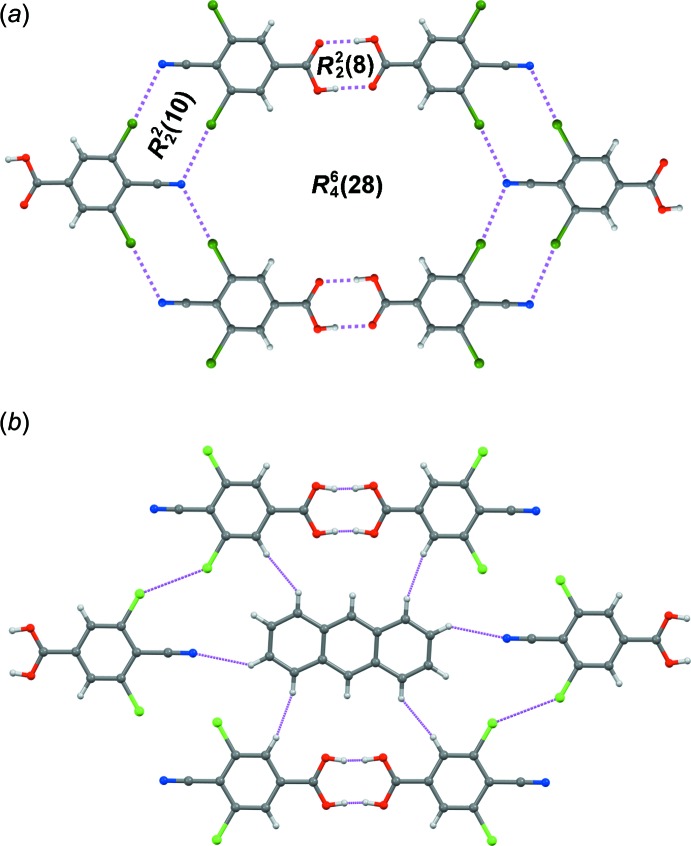
(*a*) The honeycomb-like structure envisioned by Doyle Britton. (*b*) A 2:1 co-crystal of 3,5-di­chloro-4-cyano­benzoic acid with anthracene, viewed along 28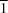 (Britton, 2012 ▸). Magenta dashed lines represent short contacts.

Doyle Britton (1930–2015) published roughly 30 crystallographic articles on solid-phase cyano–halo inter­actions from variously substituted halobenzo­nitriles and isocyanides. Britton postulated that 3,5-di­chloro-4-cyano­benzoic acid might assemble into a honeycomb-like sheet structure (Fig. 1 ▸ *a*) *via* a combination of carb­oxy hydrogen-bond dimerization and CN⋯Cl short contacts. In 2012, he found that the cyano acid mol­ecules alone do not pack in this way, but slow evaporation of mixtures containing naphthalene or anthracene afforded 2:1 acid:hydro­carbon co-crystals roughly matching his proposed structure (Britton, 2012 ▸). However, no CN⋯Cl contacts were observed (Fig. 1 ▸ *b*). Anthracene was the better fit, although it was too large to allow the ideal mol­ecular arrangement. There is no obvious substitute for anthracene or naphthalene. Thus, we have prepared anthracene co-crystals with the di­bromo analog in hopes that the larger Br bond and contact radii might close the CN⋯*X* gaps observed with Cl.
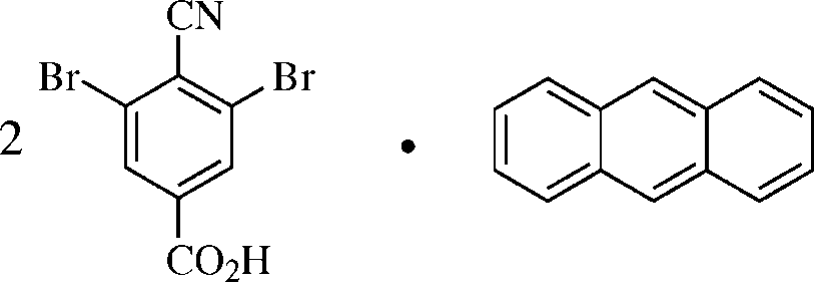


## Structural commentary   

**Figure 2:**
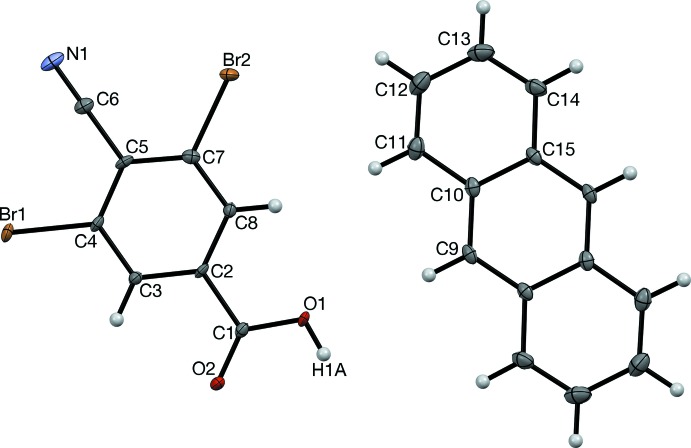
The mol­ecular structures of the components of the title co-crystal, with atom labeling and displacement ellipsoids at the 50% probability level. Only the symmetry-unique 3,5-di­bromo-4-cyano­benzoic acid mol­ecule is shown. Unlabeled anthracene atoms are generated by the (–x, –y, –z) symmetry operation.

The benzene (C2–C5/C7/C8) and anthracene (C9–C15 and symmetry equivalents, Fig. 2 ▸) ring systems are nearly planar. The mean deviation of atoms from the planes of best fit are 0.0074 (17) Å and 0.0041 (14) Å, respectively, both of which are comparable to the corresponding values in the di­chloro crystal. However, the dihedral angle between the carb­oxy group (O1–C1–O2) and the benzene ring is 3.2 (4)°, compared with 7.2° in the di­chloro analog.

## Supra­molecular features   

**Figure 3:**
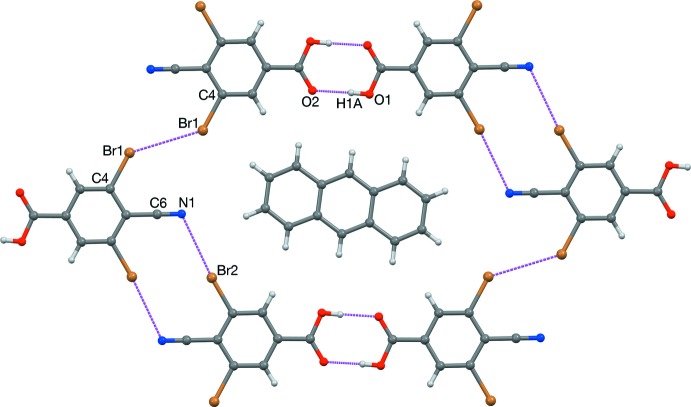
The sheet structure observed in the title co-crystal, viewed along [011].

The dihedral angle between the benzene and anthracene planes is 0.9 (2)°, which is much lower than 7.1° of the di­chloro analog. As expected, 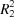
(8) carb­oxy hydrogen-bonded dimers are observed (Table 1 ▸); these are located on an inversion center. 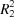
(10) rings form about another inversion center based on C6≡N1⋯Br2 contacts (Table 2 ▸); however, the corresponding N1⋯Br1 contacts are not observed (Fig. 3 ▸). Instead, 3.5534 (5) Å Br1⋯Br1 contacts form, slightly closer than the 3.70 Å non-bonded contact diameter of Br (Rowland & Taylor, 1996 ▸). In the title co-crystal, two corners of the anthracene mol­ecule contact the cyano acid network (Fig. 3 ▸), whereas all four corners made contact in the Cl analog (Fig. 1 ▸ *b*). Overall, substitution of Cl atoms with Br atoms has facilitated the formation of half of the envisioned CN⋯*X* short contacts and also improved the coplanarity of the acid and hydro­carbon mol­ecules, but anthracene is slightly too large to allow the ideal arrangement of cyano acid mol­ecules. It is possible that upon substitution of Br atoms with I atoms, the improvements would continue and the envisioned sheet structure might occur. This possibility is currently being studied in our laboratory.

## Database survey   

**Figure 4:**
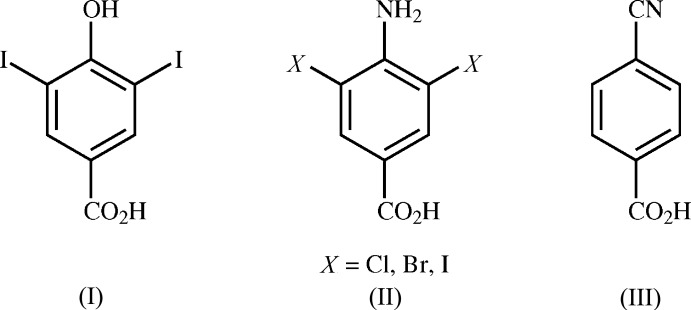
Selected structures from the database survey.

A search of the Cambridge Structural Database (Version 5.38, update May 2017; Groom *et al.*, 2016 ▸) found no 4-cyano-3-halo­benzoic acids other than the six structures reported by Britton (2012 ▸). Among 3-halo­benzoic acids, no entries were found in which carb­oxy dimers formed and assembled into a honeycomb-like sheet, with or without a co-former. Of the 40 entries for 3,5-dihalo-2,6-unsubstituted benzoic acids, 11 of them are co-crystals with carb­oxy monomers hydrogen-bonded to an O or N atom in the co-former (*i.e.*, Dubey & Desiraju, 2014 ▸; Back *et al.*, 2012 ▸). Hy­droxy acid (I) (Prout *et al.*, 1988 ▸; Fig. 4 ▸) and amino acids (II) (Pant, 1965 ▸; Ueda *et al.*, 2014 ▸) form inter­locking ribbons in which adjacent carb­oxy dimers are connected by I⋯I contacts, or amino-carb­oxy hydrogen-bonds, respectively. 4-Cyano­benzoic acid (III) forms a sheet structure in which carb­oxy dimers are connected lengthwise by 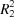
(10) rings formed by CN⋯H contacts, and laterally by 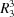
(7) rings formed by weak C—H⋯O bonds flanking each pair of carb­oxy groups (Higashi & Osaki, 1981 ▸).

## Synthesis and crystallization   

**Figure 5:**
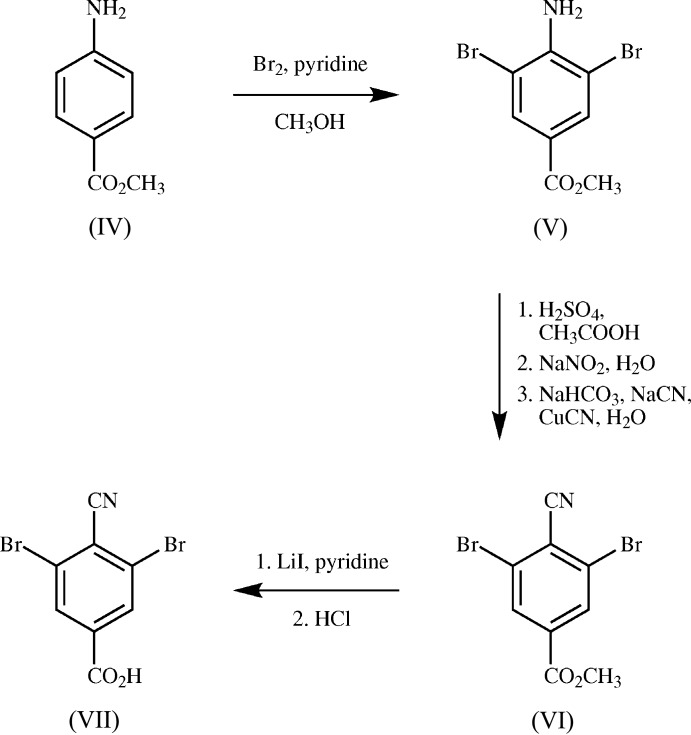
The three-step synthesis of the title cyano acid (VII).

**Methyl 4-amino-3,5-di­bromo­benzoate (V)**: Bromine (3.9 mL) and then pyridine (5.7 mL) were added dropwise to ice-cold methanol (35 mL). This mixture was added dropwise to a solution of methyl 4-amino­benzoate [(IV), commercially available, Fig. 5 ▸] in methanol (50 mL). The resulting mixture was refluxed for 4 h and then cooled to room temperature. The methanol was removed on a rotary evaporator. Di­chloro­methane (50 mL) and water (50 mL) were added. Aliquots (5 mL) of Na_2_CO_3_ solution (aq., sat.) were added until the aqueous phase remained slightly alkaline after 10 min. The organic phase was separated and then washed with Na_2_S_2_O_3_ solution (aq., sat., 25 mL), water (25 mL), brine (25 mL), and was then concentrated on a rotary evaporator. The resulting brown residue was recrystallized from ethyl acetate, giving colorless needles (18.1 g, 93%). M.p. 406–408 K (lit. 404–406 K; Otto & Juppe, 1965 ▸); ^1^H NMR (300 MHz, CDCl_3_) *δ* 8.063 (*s*, 2H), 4.996 (*s*, 2H), 3.866 (*s*, 3H); ^13^C NMR (75 MHz, CDCl_3_) *δ* 165.1 (1C), 145.9 (1C), 133.5 (2C), 121.0 (1C), 107.5 (2C), 52.3 (1C); IR (NaCl, cm^−1^) 3321, 3076, 2958, 1723, 1713, 1610, 1432, 1303, 1268, 975, 855, 761; MS (ESI, *m*/*z*) [*M*+Na]^+^ calculated for C_8_H_7_Br_2_NO_2_ 331.8715, found 331.8709.

**Methyl 3,5-di­bromo-4-cyano­benzoate (VI)**, adapted from the work of Toya *et al.* (1992 ▸): *Cyanide suspension:* NaCN (680 mg) and CuCN (480 mg) and water (40 mL) were combined in a 400 mL beaker. After the solids dissolved, NaHCO_3_ (6.5 g) was added. The resulting suspension was cooled in an ice bath. *Diazo­tization:* Di­bromo ester [(V), 720 mg] was ground in a mortar and then combined with acetic acid (2.6 mL) in a round-bottomed flask. H_2_SO_4_ (0.6 mL) was added dropwise over 1 min, followed by a solution of NaNO_2_ (313 mg) in water (1.5 mL) over 30 min.. During the course of the additions, the reaction mixture was gradually warmed in an oil bath to 315 K. *Cyanation:* When no more starting material remained, as indicated by TLC, the diazo­tization mixture was removed from the heat and then added dropwise to the cyanide suspension. The ice bath was removed. The cyanation mixture was stirred overnight and then extracted with di­chloro­methane (3 × 20 mL). The combined organic portions were washed with water (20 mL), brine (20 mL), dried with Na_2_SO_4_, filtered, and then concentrated on a rotary evaporator. The resulting brown residue was separated by column chromatography. The desired fraction (R_*f*_ = 0.34 in 3:1 hexa­ne:ethyl acetate on SiO_2_) was concentrated on a rotary evaporator, giving a tan powder (681 mg, 92%). M.p. 410–411 K; ^1^H NMR (500 MHz, CDCl_3_) δ 8.264 (*s*, 2H), 3.974 (*s*, 3H); ^13^C NMR (126 MHz, CDCl_3_) δ 163.4 (1C), 135.5 (1C), 132.6 (2C), 127.1 (2C), 122.5 (1C), 115.5 (1C), 53.5 (1C); IR (KBr, cm^−1^) 3076, 2955, 2229, 1732, 1429, 1263, 1124, 971, 748; MS (ESI, *m*/*z*) [*M*+Na]^+^ calculated for C_9_H_5_Br_2_NO_2_ 341.8559, found 341.8547.

**3,5-Di­bromo-4-cyano­benzoic acid (VII)**, adapted from the work of Lepage *et al.* (2004 ▸; especially compound 24): Cyano ester [(VI), 231 mg], lithium iodide (128 mg), and pyridine (10 mL) were combined in a round-bottomed flask. The resulting mixture was refluxed for 24 h and then cooled to room temperature. Chloro­form (25 mL), water (25 mL), and hydro­chloric acid (12 *M*, 25 mL) were added. After being stirred for 10 min, the resulting mixture was separated by suction filtration, giving a light-brown powder (217 mg, 99%). M.p. 423–425 K; ^1^H NMR (500 MHz, (CD_3_)_2_SO) *δ* 14.120 (*s*, H1*A*), 8.232 (*s*, H3*A*, H8*A*); ^13^C NMR (126 MHz, (CD_3_)_2_SO) *δ* 163.9 (C1), 137.0 (C2), 132.1 (C3, C8), 126.7 (C4, C7), 120.6 (C5), 115.9 (C6); IR (KBr, cm^−1^) 3421, 3077, 2128, 1811, 1662, 1537, 1371, 1296, 1025, 825, 770, 748; MS (ESI, *m*/*z*) [*M*–H]^−^ calculated for C_8_H_3_Br_2_NO_2_ 303.8437, found 303.8443.

**Crystallization**: 3,5-Di­bromo-4-cyano­benzoic acid (100 mg) and anthracene (29 mg) were dissolved in di­chloro­methane (25 mL) in a loosely covered beaker. Most of the solvent was allowed to evaporate gradually over 3 d. The resulting colorless or pale-orange plate-shaped crystals were collected after deca­ntation and then washed with several drops of ice-cold 1:3 di­chloro­methane:pentane.

## Refinement   

Crystal data, data collection and structure refinement details are summarized in Table 3 ▸. A direct-methods solution was calculated, followed by full-matrix least squares/difference-Fourier cycles. All H atoms were placed in calculated positions (C—H = 0.95 Å, O—H = 0.84 Å) and refined as riding atoms with *U*_iso_(H) set to 1.2*U*_eq_(C) and 1.5*U*_eq_(O).

## Supplementary Material

Crystal structure: contains datablock(s) I. DOI: 10.1107/S2056989017014815/lh5854sup1.cif

Structure factors: contains datablock(s) I. DOI: 10.1107/S2056989017014815/lh5854Isup2.hkl

Supporting information file. DOI: 10.1107/S2056989017014815/lh5854Isup3.cml

CCDC reference: 1525811

Additional supporting information:  crystallographic information; 3D view; checkCIF report

## Figures and Tables

**Table 1:** Hydrogen-bond geometry (Å, °)

*D*—H⋯*A*	*D*—H	H⋯*A*	*D*⋯*A*	*D*—H⋯*A*
O1—H1*A*⋯O2^i^	0.84	1.79	2.627 (2)	178

Symmetry code: (i) 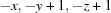.

**Table 2:** Contact geometry (Å, °)

C—*X*⋯Br	C—*X*	*X*⋯Br	C—*X*⋯Br
C6≡N1⋯Br2^ii^	1.143 (4)	3.307 (2)	115.9 (2)
C4—Br1⋯Br1^iii^	1.886 (2)	3.5534 (5)	133.43 (7)

Symmetry codes: (ii) −*x* + 2, −*y* + 1, −*z* + 2; (iii) −*x* + 1, −*y*, −*z* + 2.

**Table 3:** Experimental details

Crystal data	
Chemical formula	C_8_H_3_Br_2_NO_2_·0.5C_14_H_10_
*M*_r_	394.04
Crystal system, space group	Triclinic, *P* 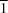
Temperature (K)	123
*a*, *b*, *c* (Å)	8.8963 (8), 9.4701 (9), 9.5839 (9)
α, β, γ (°)	115.356 (3), 106.876 (3), 94.119 (3)
*V* (Å^3^)	680.03 (11)
*Z*	2
Radiation type	Cu *K*α
μ (mm^−1^)	7.57
Crystal size (mm)	0.18 × 0.09 × 0.03

Data collection	
Diffractometer	Bruker VENTURE PHOTON-1000
Absorption correction	Multi-scan (*SADABS*; Sheldrick, 1996 ▸)
*T*_min_, *T*_max_	0.509, 0.754
No. of measured, independent and observed [*I* > 2σ(*I*)] reflections	9139, 2745, 2607
*R*_int_	0.035
(sin θ/λ)_max_ (Å^−1^)	0.625

Refinement	
*R*[*F* ^2^ > 2σ(*F* ^2^)], *wR*(*F* ^2^), *S*	0.026, 0.068, 1.06
No. of reflections	2745
No. of parameters	182
H-atom treatment	H-atom parameters constrained
Δρ_max_, Δρ_min_ (e Å^−3^)	0.40, −0.53

Computer programs: *APEX2* and *SAINT* (Bruker, 2012 ▸), *SHELXS97* (Sheldrick 2015*a* ▸), *SHELXL2014* (Sheldrick, 2015*b* ▸), *Mercury* (Macrae *et al.*, 2008 ▸) and *publCIF* (Westrip, 2010 ▸).

## supplementary crystallographic information

### Crystal data

**Table:**

C_8_H_3_Br_2_NO_2_·0.5C_14_H_10_	*Z* = 2
*M**_r_* = 394.04	*F*(000) = 382
Triclinic, *P*1	*D*_x_ = 1.924 Mg m^−^^3^
*a* = 8.8963 (8) Å	Cu *K*α radiation, λ = 1.54178 Å
*b* = 9.4701 (9) Å	Cell parameters from 2967 reflections
*c* = 9.5839 (9) Å	θ = 5.3–74.6°
α = 115.356 (3)°	µ = 7.57 mm^−^^1^
β = 106.876 (3)°	*T* = 123 K
γ = 94.119 (3)°	Plate, pale orange
*V* = 680.03 (11) Å^3^	0.18 × 0.09 × 0.03 mm

### Data collection

**Table:**

Bruker VENTURE PHOTON-1000 diffractometer	2607 reflections with *I* > 2σ(*I*)
φ and ω scans	*R*_int_ = 0.035
Absorption correction: multi-scan (SADABS; Sheldrick, 1996)	θ_max_ = 74.6°, θ_min_ = 5.3°
*T*_min_ = 0.509, *T*_max_ = 0.754	*h* = −11→11
9139 measured reflections	*k* = −11→11
2745 independent reflections	*l* = −11→11

### Refinement

**Table:**

Refinement on *F*^2^	0 restraints
Least-squares matrix: full	Hydrogen site location: inferred from neighbouring sites
*R*[*F*^2^ > 2σ(*F*^2^)] = 0.026	H-atom parameters constrained
*wR*(*F*^2^) = 0.068	*w* = 1/[σ^2^(*F*_o_^2^) + (0.0369*P*)^2^ + 0.5603*P*] where *P* = (*F*_o_^2^ + 2*F*_c_^2^)/3
*S* = 1.06	(Δ/σ)_max_ = 0.001
2745 reflections	Δρ_max_ = 0.40 e Å^−^^3^
182 parameters	Δρ_min_ = −0.53 e Å^−^^3^

### Special details

**Table:**

Geometry. All esds (except the esd in the dihedral angle between two l.s. planes) are estimated using the full covariance matrix. The cell esds are taken into account individually in the estimation of esds in distances, angles and torsion angles; correlations between esds in cell parameters are only used when they are defined by crystal symmetry. An approximate (isotropic) treatment of cell esds is used for estimating esds involving l.s. planes.

### Fractional atomic coordinates and isotropic or equivalent isotropic displacement parameters (Å^2^)

**Table:**

	*x*	*y*	*z*	*U*_iso_*/*U*_eq_	
Br1	0.38562 (3)	0.12356 (3)	0.93908 (3)	0.01483 (9)	
Br2	0.81803 (2)	0.51847 (3)	0.84679 (3)	0.01686 (9)	
O1	0.21809 (18)	0.54610 (19)	0.5615 (2)	0.0154 (3)	
H1A	0.1338	0.5671	0.5146	0.023*	
O2	0.04198 (18)	0.38413 (18)	0.5858 (2)	0.0139 (3)	
N1	0.8230 (2)	0.2641 (3)	1.0526 (3)	0.0211 (4)	
C1	0.1800 (3)	0.4429 (2)	0.6097 (3)	0.0102 (4)	
C2	0.3217 (2)	0.4018 (2)	0.7011 (2)	0.0092 (4)	
C3	0.2929 (2)	0.2999 (2)	0.7650 (2)	0.0096 (4)	
H3A	0.1859	0.2573	0.7495	0.011*	
C4	0.4229 (3)	0.2615 (2)	0.8516 (2)	0.0100 (4)	
C5	0.5815 (2)	0.3249 (2)	0.8767 (2)	0.0102 (4)	
C6	0.7162 (3)	0.2888 (3)	0.9728 (3)	0.0135 (4)	
C7	0.6057 (3)	0.4265 (3)	0.8107 (3)	0.0116 (4)	
C8	0.4777 (3)	0.4647 (2)	0.7223 (2)	0.0103 (4)	
H8A	0.4958	0.5328	0.6766	0.012*	
C9	0.4142 (3)	0.8993 (2)	0.5372 (3)	0.0131 (4)	
H9A	0.3562	0.8307	0.5622	0.016*	
C10	0.5827 (3)	0.9268 (2)	0.5908 (3)	0.0126 (4)	
C11	0.6714 (3)	0.8552 (3)	0.6831 (3)	0.0179 (5)	
H11A	0.6149	0.7875	0.7102	0.022*	
C12	0.8344 (3)	0.8823 (3)	0.7326 (3)	0.0230 (5)	
H12A	0.8912	0.8345	0.7947	0.028*	
C13	0.9206 (3)	0.9822 (3)	0.6915 (3)	0.0247 (5)	
H13A	1.0347	0.9989	0.7249	0.030*	
C14	0.8420 (3)	1.0540 (3)	0.6055 (3)	0.0198 (5)	
H14A	0.9019	1.1211	0.5803	0.024*	
C15	0.6700 (3)	1.0296 (2)	0.5522 (3)	0.0121 (4)	

### Atomic displacement parameters (Å^2^)

**Table:**

	*U*^11^	*U*^22^	*U*^33^	*U*^12^	*U*^13^	*U*^23^
Br1	0.01625 (14)	0.01597 (13)	0.01776 (14)	0.00482 (9)	0.00336 (10)	0.01426 (10)
Br2	0.00653 (13)	0.02493 (15)	0.02190 (14)	0.00266 (9)	0.00341 (10)	0.01464 (11)
O1	0.0098 (7)	0.0197 (8)	0.0244 (8)	0.0037 (6)	0.0021 (6)	0.0196 (7)
O2	0.0079 (7)	0.0163 (7)	0.0211 (8)	0.0027 (6)	0.0017 (6)	0.0141 (6)
N1	0.0163 (10)	0.0255 (10)	0.0221 (10)	0.0110 (8)	0.0021 (8)	0.0136 (9)
C1	0.0104 (10)	0.0099 (9)	0.0095 (9)	0.0028 (7)	0.0010 (7)	0.0053 (7)
C2	0.0092 (9)	0.0086 (9)	0.0081 (9)	0.0037 (7)	−0.0007 (7)	0.0046 (7)
C3	0.0084 (9)	0.0091 (9)	0.0107 (9)	0.0020 (7)	0.0014 (7)	0.0054 (7)
C4	0.0115 (10)	0.0100 (9)	0.0090 (9)	0.0045 (7)	0.0011 (8)	0.0062 (8)
C5	0.0080 (9)	0.0111 (9)	0.0082 (9)	0.0045 (7)	−0.0005 (7)	0.0035 (8)
C6	0.0117 (10)	0.0140 (10)	0.0131 (10)	0.0048 (8)	0.0025 (8)	0.0059 (8)
C7	0.0088 (9)	0.0128 (9)	0.0112 (9)	0.0016 (7)	0.0028 (8)	0.0044 (8)
C8	0.0113 (9)	0.0095 (9)	0.0108 (9)	0.0021 (7)	0.0025 (8)	0.0063 (8)
C9	0.0162 (10)	0.0082 (9)	0.0148 (10)	0.0001 (8)	0.0070 (8)	0.0047 (8)
C10	0.0172 (11)	0.0081 (9)	0.0113 (9)	0.0016 (8)	0.0056 (8)	0.0033 (8)
C11	0.0245 (12)	0.0125 (10)	0.0156 (10)	0.0033 (9)	0.0049 (9)	0.0069 (9)
C12	0.0257 (13)	0.0167 (11)	0.0196 (11)	0.0055 (9)	−0.0010 (10)	0.0082 (9)
C13	0.0135 (11)	0.0222 (12)	0.0276 (13)	0.0018 (9)	0.0007 (9)	0.0071 (10)
C14	0.0131 (11)	0.0152 (11)	0.0261 (12)	−0.0012 (8)	0.0052 (9)	0.0072 (9)
C15	0.0138 (10)	0.0073 (9)	0.0131 (9)	0.0000 (7)	0.0057 (8)	0.0027 (8)

### Geometric parameters (Å, º)

**Table:**

Br1—C4	1.886 (2)	C8—H8A	0.9500
Br2—C7	1.890 (2)	C9—C15^i^	1.394 (3)
O1—C1	1.309 (3)	C9—C10	1.400 (3)
O1—H1A	0.8400	C9—H9A	0.9500
O2—C1	1.224 (3)	C10—C11	1.432 (3)
N1—C6	1.142 (3)	C10—C15	1.432 (3)
C1—C2	1.493 (3)	C11—C12	1.356 (4)
C2—C3	1.390 (3)	C11—H11A	0.9500
C2—C8	1.391 (3)	C12—C13	1.423 (4)
C3—C4	1.386 (3)	C12—H12A	0.9500
C3—H3A	0.9500	C13—C14	1.359 (4)
C4—C5	1.402 (3)	C13—H13A	0.9500
C5—C7	1.394 (3)	C14—C15	1.433 (3)
C5—C6	1.446 (3)	C14—H14A	0.9500
C7—C8	1.381 (3)	C15—C9^i^	1.394 (3)
			
C1—O1—H1A	109.5	C2—C8—H8A	120.5
O2—C1—O1	124.6 (2)	C15^i^—C9—C10	121.3 (2)
O2—C1—C2	121.29 (19)	C15^i^—C9—H9A	119.4
O1—C1—C2	114.08 (18)	C10—C9—H9A	119.4
C3—C2—C8	121.22 (19)	C9—C10—C11	122.1 (2)
C3—C2—C1	117.98 (18)	C9—C10—C15	119.3 (2)
C8—C2—C1	120.80 (19)	C11—C10—C15	118.6 (2)
C4—C3—C2	118.88 (19)	C12—C11—C10	121.2 (2)
C4—C3—H3A	120.6	C12—C11—H11A	119.4
C2—C3—H3A	120.6	C10—C11—H11A	119.4
C3—C4—C5	121.09 (19)	C11—C12—C13	120.0 (2)
C3—C4—Br1	119.34 (16)	C11—C12—H12A	120.0
C5—C4—Br1	119.57 (15)	C13—C12—H12A	120.0
C7—C5—C4	118.42 (19)	C14—C13—C12	121.0 (2)
C7—C5—C6	121.09 (19)	C14—C13—H13A	119.5
C4—C5—C6	120.47 (19)	C12—C13—H13A	119.5
N1—C6—C5	178.1 (2)	C13—C14—C15	120.7 (2)
C8—C7—C5	121.37 (19)	C13—C14—H14A	119.7
C8—C7—Br2	119.04 (16)	C15—C14—H14A	119.7
C5—C7—Br2	119.57 (16)	C9^i^—C15—C10	119.5 (2)
C7—C8—C2	119.02 (19)	C9^i^—C15—C14	122.1 (2)
C7—C8—H8A	120.5	C10—C15—C14	118.5 (2)
			
O2—C1—C2—C3	−2.9 (3)	Br2—C7—C8—C2	−177.60 (15)
O1—C1—C2—C3	176.48 (18)	C3—C2—C8—C7	−1.0 (3)
O2—C1—C2—C8	177.60 (19)	C1—C2—C8—C7	178.55 (18)
O1—C1—C2—C8	−3.1 (3)	C15^i^—C9—C10—C11	179.81 (19)
C8—C2—C3—C4	0.2 (3)	C15^i^—C9—C10—C15	−0.3 (3)
C1—C2—C3—C4	−179.35 (18)	C9—C10—C11—C12	179.4 (2)
C2—C3—C4—C5	0.6 (3)	C15—C10—C11—C12	−0.5 (3)
C2—C3—C4—Br1	179.97 (14)	C10—C11—C12—C13	−0.6 (4)
C3—C4—C5—C7	−0.7 (3)	C11—C12—C13—C14	1.2 (4)
Br1—C4—C5—C7	180.00 (14)	C12—C13—C14—C15	−0.7 (4)
C3—C4—C5—C6	177.55 (18)	C9—C10—C15—C9^i^	0.3 (3)
Br1—C4—C5—C6	−1.8 (3)	C11—C10—C15—C9^i^	−179.81 (19)
C4—C5—C7—C8	−0.1 (3)	C9—C10—C15—C14	−178.88 (19)
C6—C5—C7—C8	−178.35 (19)	C11—C10—C15—C14	1.0 (3)
C4—C5—C7—Br2	178.40 (14)	C13—C14—C15—C9^i^	−179.6 (2)
C6—C5—C7—Br2	0.2 (3)	C13—C14—C15—C10	−0.4 (3)
C5—C7—C8—C2	1.0 (3)		

Symmetry code: (i) −*x*+1, −*y*+2, −*z*+1.

### Hydrogen-bond geometry (Å, º)

**Table:**

*D*—H···*A*	*D*—H	H···*A*	*D*···*A*	*D*—H···*A*
O1—H1*A*···O2^ii^	0.84	1.79	2.627 (2)	178

Symmetry code: (ii) −*x*, −*y*+1, −*z*+1.
